# On Pulmonary Consumption

**Published:** 1814-05

**Authors:** Richard Walker

**Affiliations:** Oxford


*For the Medical and Physical Journal.*

### *On Pulmonary Consumption; by* Mr. Richard Walker, of Oxford.

(Continued from p. 302.)

DISMISSING, then, the *visionary idea* of curing, or even relieving this disease, by any *medicine* supposed to have the power of acting *specifically* upon it; which, however amusing in theory to the inexperienced, never has been, I am certain, and I venture to predict never will be, confirmed by FACT; I shall proceed to the only rational means by which this destructive malady is to be treated with any prospect of success. I shall confine myself, here, to the *organic pulmonary consumption*, which ordinarily originates in a constitutional acrimony in the habit, either of a scrofulous or scorbutic nature, which most usually, in consequence of some exciting cause, as catching cold, as it is called, fixes on the lungs, commencing in tubercles, proceeding on to abscesses, and terminating in ulcers.

The mode of *preventing* the disease, in such habits as are predisposed to it, or *curing* it when formed, must therefore depend, chiefly, upon the means recommended for the cure of those in whom, with the same diseased habit, but from a differently exciting cause, the disease fixes and vents itself in some other part, viz. by an artificial outlet, made sufficiently early, in a convenient situation, by way of draining from the habit the noxious humor, and thus drawing it off, off, or diverting it from the lungs; observing to avoid or provide against, as much as possible, every exciting cause, viz. the effects of cold and moisture in the atmosphere; heating and stimulating nourishment; and strong liquors of every description: and using medicinally antiphlogistics, and anodynes occasionally, so regulated as to suspend the cough, and procure rest, without acting as a stimulant: for this intention, *opium*, in a solid form, is fittest, exhibited at due intervals, and in a dose sufficiently small as to act rather as a sedative than as a stimulant; with demulcent liquors and lubricants, to allay the irritation at the trachea, and thereby prevent the mischief, as much as possible, arising to the lungs from coughing. If the disease, before application, has advanced so far as that the patient expectorates purulent matter in considerable quantity, and is evidently sinking under the disease, recourse must be had to a more nutritious restorative diet, with cordials and anodynes, obviating as much as possible to the last every distressing symptom and circumstance, by the most appropriate means, both of diet and medicine. A uniform temperature is of so much consequence in this complaint, that I am convinced, if patients, in a recent state of this disease, could be removed to a climate where the temperature is constantly about 64 or 65, with the above management, and without being annoyed by a pretended specific, it would much seldomer prove fatal. The salutary introduction of the use of flannel to the skin and socks to the feet, to all tender persons, especially such as are particularly liable to affections of the chest, rheumatisms, &c. must be so obvious now as never to be overlooked.

Hence, the only mode to be pursued in *pulmonary consumption*, for which there is certainly no direct remedy in nature, the means excepted before pointed out, when evidently arising from a scrofulous cause, or more particularly what I have denominated a scorbutic-scrofulous affection;* and whilst in an incipient state, at which time alone a cure can be reasonably expected, is to allay the irritation in the lungs, abate the hectic fever, and at the same time to support a due degree of vigor in the habit. No medicine in nature has any specific effect in abating or suspending the cough arising from the irritation in the lungs, in consequence of disease, except opium, (premising blood-letting occasionally, as circumstances may indicate.) The due administration, therefore, of opium, at proper intervals, with

* This alludes to the article *Scrofula*, and which will probably form the subject of my next paper.

the mechanical effects of medicines of an emollient lubricating quality,* to allay, temporarily, the irritation in the trachea, the immediate cause of the cough, are the only appropriate medicines for this intention. The hectic fever being the effect of the diseased affection of the lungs, demands our attention in a secondary way only, and for which, in addition to the former, such medicines as are congenial to the inflamed irritable state of the lungs may be administered, as refrigerants, antiphlogistics, &c.

Since the effects upon the lungs of violent and repeated coughing are most to be dreaded, which by rending or lacerating the lungs may induce hæmorrhage, or hasten or bring on suppuration, every means should be used to allay or prevent this. In addition, therefore, to what has been observed before, particular care should be taken that the patient be supported by a bland, cooling, nutritious, lubricating diet, exhibited at shorter intervals than is usual in a state of health; for the patient is not only injured more by any incidental fits of coughing which occur, but is also much more susceptible or liable to coughing during a state of debility, than in a due state of vigor of body; and, however trifling it may appear, it is of the utmost importance, to save the lungs, by exerting every innate effort of suppressing the cough, as much as possible, instead of giving way to it, as is the ordinary way, upon every irritation or excitation to coughing, and which may be effected, by due exertion, in a very considerable degree, and with eminent advantage. By this means, when such irritation on the trachea, and excitation to coughing, has occurred to myself in the night, to a most distressing degree, at a time I was unprepared for it, I have entirely, or in a great degree, obviated the ill effects I had formerly experienced on such occasions, merely by collecting in my mouth my saliva, in quantity, and then swallowing it, in order to supply the place of other lubricants; and exerting my utmost efforts, and very effectually, in obviating to a considerable degree the distress of coughing.

In order to appease the cough, especially during the night, in pulmonary consumption, as well as catarrh, re-

* The lubricating medicines, ordinarily administered, consist of the mucilages of gum acacia, gum tragacanth, quince-seed, or linseed; of which, perhaps, the last is most efficacious, exhibited according to the formula in the last London Pharmacopœia, viz. the infusum lini; the decoct. hord. comp. likewise, with the addition of gum acacia, is a very good formula.

course course must be had to a prudent administration of opium; and the occasional, and in some cases the almost incessant administration of lubricants, which (in the case of consumption, where little nutriment is taken,) should be likewise of an innocently nutritive nature, thus answering at the same time two different intentions, viz. appeasing the cough and supporting the patient, avoiding every thing likewise of an oily or unctuous nature.

So essential are lubricants for appeasing cough, and removing or obviating irritation in the trachea, which for want of them is sometimes extremely distressing, that when no liquor of this nature is at hand, as might happen in the night, even the saliva itself, as I have observed before, collected for the purpose of swallowing as a substitute, as I have frequently experienced myself, will remove the most distressing sensations from this cause, and avert an incessant coughing the whole night. Persons in general are too apt to indulge in, or give way to, every excitement in the trachea; much however may be done, and effectively, by exerting proper efforts to suppress and put aside the cough, and by this means the lungs, already in a tender and irritable state, prevented from much injurious and unnecessary exertion, and any expectoration which is unavoidable brought up much easier and more perfectly.

Ordinary persons have a notion that affections of the chest are seated in the stomach, as they term it; or that whatever medicine is taken goes immediately to the seat of the disease. This erroneous opinion, being productive of evil consequences, should ever be removed; and they should be informed that the efficacy of lubricants, simply so considered, only act by lubricating the parts, as they pass over in their way to the stomach; and that it is not the quantity that is swallowed, but the frequency of the application, which produces the wished-for effect.

A certain author, speaking of the trifling light in which a cough is considered by many persons, says, "more people die in this country of cough than any other disease; which in its commencement might have been readily cured by the most simple medicines." I am perfectly assured of the truth of this observation; and I might add, that of those who are placed under the care of medical persons of reputed eminence in their profession, many die which might have been saved by means which are entirely despised by the patient and attendants, and most generally neglected or disregarded by the physician.

Had I not been of the medical profession, and with such advantages advantages of experience as to know how little reliance is to be placed in the usual mode or routine of practice in such a complaint, and of the total inefficacy of the medicines ordinarily used in such cases, I might, I am of opinion, in consequence of cough, during winter seasons, have died a dozen times or more, had I been possessed of so many lives; whereas I have hitherto preserved my life from this and various other threatening illnesses, by the means which I have had occasion to notice in different parts of this work, but which, unfortunately for other persons under similar circumstances, I apprehend will not obtain that credit which I well know to be due to it, or that they will appreciate its real value.

In affections of the chest, as peripneumony, pleurisy, and in catarrh, cough, &c. especially if the person be disposed to it, the first question asked should be, whether the patient wears a flannel waistcoat; and if not, it is the first thing to be insisted on. I have cured many patients by this means alone, whom it would have been impossible to have convinced were not cured by the medicines administered to them, at the same time one particle of which, under similar circumstances, I would not have taken myself. The use of flannel is equally applicable in rheumatic affections; and the constant use of a flannel waistcoat, after once commencing it, viz. in the middle time of life, or earlier, if a tenderness of habit require it, should never afterwards be omitted.

I am convinced almost every instance of pulmonary consumption might be averted, by attention to this circumstance, and others of a similar tendency, which I have mentioned, if adopted sufficiently early.

I may venture to say, without any fear of contradiction, that the introduction of under flannel waistcoats, which is but of late date, although now almost universally used, has been more beneficial to the community at large, respecting the preservation of life and health, than any discovery in the medical profession which has been made during a century back from the present time.*

I shall have occasion hereafter, when speaking of *Noli me tangere*, an affection which I consider to be of a scrofulous nature, to notice the case of a young woman who was admitted into the Infirmary for the cure of this complaint, in

* *Vaccination*, it might be said, is an exception to this observation; but this, I think, is not strictly applicable to the present circumstance.

other respects perfectly well. After a few months, (not in consequence, apparently, of any applications, which, in this instance, were merely of a mild or palliative nature,) the sore healed, she became consumptive, and died, without leaving the Infirmary. In this instance the disease evidently became transferred to the lungs. I have no doubt, in this instance, that an artificial outlet, in the manner before alluded to, properly managed, (which, by the bye, as I shall have occasion to show hereafter, is much more difficult to manage effectively than is ordinarily supposed,) would have rendered the cure of the original complaint easy and safe, and obviated or prevented the affection on the lungs.

The following observations on *Regimen* and *Clothing* forming an appropriate appendage to the subject of this paper, I shall subjoin them.

Regimen and Clothing.—A strict attention to each of these is in reality of the first importance towards recovery from almost all indispositions, and from many diseases, as well as for the preservation of health; but which are certainly not sufficiently regarded by many practitioners, and almost entirely despised by the sick and their attendants.

The reason of this, in the first place, arises from the practitioner not having reached the perfection of his art, viz. not having ascertained the defined limits of the efficacy of medicine in certain cases, and the almost indefinite extent of the efficacy of a judicious regimen; secondly, because such doctrine, though strictly true, being utterly incomprehensible to persons out of the profession, would be attributed to ignorance, excepting by such persons as unfortunately have experienced much serious illness, and in consequence have learned how to appreciate the value of it. In fact, there are very few diseases indeed that are curable by medicines, independently of a due attention to regimen; but there are a great many diseases and indispositions which are removable by an appropriate regimen alone. An experienced practitioner will frequently rely himself chiefly upon the salutary effects of the regimen he directs; whilst the patient reluctantly adheres to his regimen, but swallows his physic with eagerness; and when the cure is completed by the most appropriate means, it is of no consequence to which the credit is due; nor is the physician less deserving of his reward. This knowledge is of the first importance in the treatment of chronic rheumatism, coughs, consumptions, &c. which commonly originate from a neglect of attention to one or other of these matters, and for which complaints there there being certainly no direct remedy in medicine, too much attention cannot be paid. A flannel under waistcoat, and warm socks to the feet, are at once the best preservative against coughs, chronic rheumatisms, &c. as well as remedy for them when present. Moreover, regimen, or diet, persevered in for a length of time, in many instances effects a perfect change in the constitution, merely by introducing into or filling the habit with more wholesome juices, in the stead of those which were derived by improper nutriment; and by that means many diseases, especially such as depend upon acrimony in the habit, as *Scrofula*, (which is the parent of so many diseases,) *Scurvy*, (commonly so called,) *Gout*, &c. may be mitigated, if not entirely removed; hence nothing can be more absurd than trusting to any medicine in these diseases, whilst, as is too often the case, a regimen which produces or constantly feeds the disease is persisted in. It is upon this principle, I apprehend, that *diet drinks*, consisting of the juices of bland and innocent herbs, and other substances of a similar nature, more than in any specific qualities they may possess, are beneficial in such cases.

